# LRRK2 inhibition potentiates PARP inhibitor cytotoxicity through inhibiting homologous recombination‐mediated DNA double strand break repair

**DOI:** 10.1002/ctm2.341

**Published:** 2021-02-26

**Authors:** Lifeng Chen, Jing Hou, Xiangyu Zeng, Qiang Guo, Min Deng, Jake A Kloeber, Xinyi Tu, Fei Zhao, Zheming Wu, Jinzhou Huang, Kuntian Luo, Wootae Kim, Zhenkun Lou

**Affiliations:** ^1^ Department of Gynecology Zhejiang Provincial People's Hospital Hangzhou Zhejiang P. R. China; ^2^ Department of Molecular Pharmacology and Experimental Therapeutics Mayo Clinic Rochester Minnesota; ^3^ Department of Oncology, Medical Scientist Training Program Mayo Clinic Rochester Minnesota

**Keywords:** HR, LRRK2 inhibitor, PARP inhibitor, Rad51

## Abstract

PARP inhibitors induce DNA lesions, the repair of which are highly dependent on homologous recombination (HR), and preferentially kill HR‐ deficient cancers. However, cancer cells have developed several mechanisms to transform HR and confer drug resistance to PARP inhibition. Therefore, there is a great clinical interest in exploring new therapies that induce HR deficiency (HRD), thereby sensitizing cancer cells to PARP inhibitors. Here, we found that GSK2578215A, a high‐selective and effective leucine‐rich repeat kinase 2 (LRRK2) inhibitor, or LRRK2 depletion suppresses HR preventing the recruitment of RAD51 to DNA damage sites through disruption of the interaction of RAD51 and BRCA2. Moreover, LRRK2 inhibition or depletion increases the susceptibility of ovarian cancer cells to Olaparib in vitro and in vivo. In clinical specimens, LRRK2 high expression is high related with advanced clinical characteristics and poor survival of ovarian cancer patients. All these findings indicate ovarian cancers expressing high levels of LRRK2 are more resistant to treatment potentially through promoting HR. Furthermore, combination treatment with an LRRK2 and PARP inhibitor may be a novel strategy to improve the effectiveness of LRRK2 expression ovarian cancers.

## Graphical abstract

LRRK2 inhibitor GSK2578215A suppresses HR and sensitizes ovarian cancer cells to PARP inhibitor.

LRRK2 inhibition impedes the recruitment of RAD51 by disrupting the interaction of RAD51 and BRCA2.

High expression of LRRK2 is associated with poor survival of ovarian cancer patients.

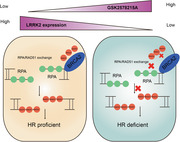

## 1 INTRODUCTION

Ovarian cancer (OC) is one of the most general tumors responsible for mortality in women. It is the most lethal malignancy to destroy the female reproductive system. Cytoreductive surgery in combination with platinum‐based chemotherapy remains the standard therapeutic strategy for OC. Despite initial aggressive response, the majority of patients become less responsive accompanied by subsequent relapses, leading to a poor 5‐year relative survival rate of 15‐30% for advanced‐stage OC.^1^ Advanced OCs often exhibit increased genome instability, most of which harbor defects in DNA damage response (DDR) and repair. And approximately 13% of these patients carry a mutation in genes involved homologous recombination (HR) pathway, such as breast cancer gene 1/2 (BRCA1/2).^2^ HR is important for error‐free DNA double‐strand break repair, thereby maintaining genome stability.^3^ Although HR deficiency (HRD) induces genome instability and potentiates tumorigenesis, this defect has provided an opportunity for therapeutic use in treating cancers. Poly (ADP‐ribose) polymerase inhibitors (PARPi) (Niraparib, Rucaparib, and Olaparib) recently approved by the FDA have been applied to OC patients bearing BRCA1/2 gene mutations or those sensitive to platinum‐based chemotherapeutic agents. PARP inhibition (PARPi) blocks the activity of PARP, leading to DNA lesions and subsequent double strand breaks (DSBs), the repair of which is highly dependent on HR.^4^ Therefore, PARPi preferentially kills HR‐deficient cancers. Similar to platinum therapy, cancer cells have developed several mechanisms to adjust HR and confer therapeutic resistance to PARP inhibitor.^5^, ^6^.Overall, inducing HRD is a feasible strategy to increase sensitivity of cells to DNA damaged therapies in both HR‐deficient and ‐proficient cancers. Here, we screened a library of small molecules to identify potential drugs that suppress HR and increase susceptibility of OC cells to PARPi therapy. We presented that GSK2578215A sensitizes LRRK2 high expression OC cells to Olaparib in vitro and in vivo. Mechanically, it suppresses HR by preventing RAD51 from recruiting to DNA damage sites via disrupting the interaction of RAD51 and BRCA2. As target of GSK2578215A, LRRK2 was proved to be important and plays important role in HR in this study. LRRK2 belongs to the leucine‐rich repeat kinase family, which is widely expressed in brain, kidney, and lung. It is considered to be related to familial Parkinson's disease (PD),^7^ tuberculosis,^8^ inflammatory bowel disease,^9^ and leprosy. Recently, the role of LRRK2 in cancer has received considerable attention. Emerging evidence suggests that LRRK2 mutations are highly related with cancers such as colon cancer and nonskin cancer.^10^, ^11^ Article also reported that LRRK2 is overexpressed in thyroid cancer.^12^ Here, we noticed that in OC patients, LRRK2 high expression is associated with advanced clinical factors. All the findings indicate that OCs expressing high levels of LRRK2 are more resistant to treatment potentially through promoting the HR pathway. Furthermore, combination treatment with an LRRK2 inhibitor and a PARP inhibitor may be a novel strategy to improve treatment effectiveness in OCs with high expression of LRRK2.

## 2 MATERIALS AND METHODS

### 2.1 Cell culture, antibodies, and plasmids

The human U2OS, HEK293T, OVCAR8, and OVCAR10 were purchased from ATCC. PEO1 and OV56 were bought from Sigma. OVKATE cells purchased from JCRB Cell Bank. Cells were immersed in McCoy's 5A, Dulbecco's Modified Eagle's Medium (DMEM), or RPMI1640, or added with 10% fetal bovine serum (FBS) in 5% CO2 at 37°C. pDEST51‐LRRK2 was purchased from Addgene (#25080). Full‐length LRRK2 was constructed into pLVX3 lentiviral vector and then lentivirus was packaged, and OVCAR10 cells were infected with the virus and selected with puromycin for 10 days to establish stable cell lines. pCMV‐Myc‐RAD51 was given by Dr. Junjie Chen (University of Texas MD Anderson Cancer Center). pCHMWS‐FLAG‐LRRK2 and mutations (K1906M,G2019S, and pS935/910) were gifts from Dr. Cookson (Molecular Genetics Section, Laboratory of Neurogenetics, National Institute on Aging). Anti‐RAD51 (GTX100469) antibody was purchased from GeneTex. Anti‐BRCA1 (SC‐6954), Antigreen fluorescent protein [GFP] (SC‐9996), Anti‐RPA2 (SC‐56770), and Anti‐Myc (sc‐40, mouse) antibodies were sold by Santa Cruz Biotechnology. Anti‐BRCA2 (pS3291,AB9986) was bought from sigma. Anti‐BRCA2 (OP95‐100UG) was purchased from Millipore Anti‐V5 (SV5‐Pk1) antibody, Anti‐LRRK2(pS910)(ab133450), and Anti‐LRRK2(pS935)(ab133449) were purchased from Abcam.

### 2.2 SeeSaw 2.0 reporter assay

In the SeeSaw reporter SSR 2.0 system, two I‐SceI target sites in opposite orientation were cloned at the 3‐end of the green fluorescent protein (GFP) gene. I‐SceI expression induces a DSB; when damage is repaired by nonhomologous end joining (NHEJ), cells will express GFP, while damage is repaired by HR, cells will express red fluorescent protein (RFP).^13^ In this study, we used OVCAR8 cells harboring the SeeSaw 2.0 reporter system to analyze the effectiveness of DSB repair. OVCAR8 cells were transfected with SeeSaw 2.0 according to protocol and selected using G418 for 2 weeks. After selection, the cells were infected with the lentivirus expressing I‐SceI and seeded into 96‐wells plates at a density of 1000 cells/well after 6 hours. Cells were treated with indicated drugs after 24 h. Thirty‐six hours later, cells were harvested and fixed with 4% paraformaldehyde for 20 min at RT, rinsed with PBS, and then subjected to flow cytometric analysis (Thermo fisher, Attune NxT Flow cytometer).

### 2.3 HR and NHEJ reporter assay

HEK293T cells were plated in 24‐well plates. Cells were transfected with HR reporter (DR‐GFP) or NHEJ reporter (EJ5‐GFP) along with pCBA‐I‐SceI and mCherry after 24 h. Forty‐eight hours later, cells were harvested and then subjected to flow cytometric analysis (Thermo fisher).

### 2.4 Western blot and immunoprecipitation

Harvested cells were lysed with NETN buffer (20 mM Tris‐HCl [pH 8.0], 0.1 mM EDTA, 100 mM NaCl, 0.5% NP‐40 with 10 mM NaF, protease inhibitors) for 30 min before centrifugation. Then supernatants were immunoprecipitated with indicated agarose beads (overnight, 4°C). The immunoprecipitates were rinsed using NETN three times before centrifugation. A sum of 30 μL supernatant was then added with 1× Laemmli buffer subjected to SDS–PAGE separation before Immunoblotting was performed. The quantification of Western Blots was performed by ImageJ.

### 2.5 Cell cycle assays

Cells were dissociated and then fixed in 70% cooled ethanol (overnight, −20°C) and incubated with propidium iodide (PI) supplemented with RNase (30 min RT). Flow cytometry (Thermo fisher, Attune NxT Flow cytometer) and ModFit LT software were used for analyzing cell cycle.

### 2.6 Colony formation analysis

A sum of 500‐800 cells were seeded in six‐well plates. Cells were treated with different treatment strategies after 24 h. After 12‐14 days, colonies were fixed with methanol (30 min, RT), stained with 0.1% Giemsa (10 min, RT) and quantified.

### 2.7 Immunofluorescence staining

Immunofluorescence staining was carried out following a standard process. Briefly, cells were plated into coverslips. For BRCA1 foci, cells were fixed in 3% paraformaldehyde for 20 min, and then permeabilized in 0.5% Triton‐X (5 min, RT). For RAD51 foci, cells were permeabilized with 0.5% Triton‐X for 5 min at 4°C, washed with PBS, and then fixed in 4% paraformaldehyde for 20 min. For RPA2 foci, cells were fixed with methanol:acetone (1:1) at −20°C for 25 min, rinsed by PBS, and then fixed in 4% paraformaldehyde for 20 min. After washing with PBS, cells were then incubated with primary antibody (overnight, 4°C). Next, cells were washed by PBS three times, and then incubated with secondary antibodies (30 min, 37°C). DAPI was used to counterstain Nuclei. Cells were fixed with antifading solution. The number of foci were visualized and counted using ImageXpress confocal high‐content imaging system (Molecular device).

### 2.8 ShRNA and sgRNA

LRRK2 shRNAs were purchased from Sigma: LRRK2 shRNA‐1: CGCAGCTTTCAGCGATTCTAA; LRRK2 shRNA‐2: TCCACTTTGCAGCGCTTTAAA. OVCAR8 Knockout cells were generated using CRISPR. Briefly, two LRRK2 sgRNAs, GAGTCCAAGACGATCAACAG and AACGCTGGTCCAAATCCTGG, were inserted into LentiCRISPRv2 (Addgene). Lentivirus was packaged, and cells were infected with the virus and selected with puromycin for 10 days to establish stable cell line.

### 2.9 Tumor xenograft

The experiments were completed with the approval of the Committee for the Care and Use of Animals at the Mayo Clinic (Rochester, MN). Five‐week old female athymic nude Ncr nu/nu mice were performed subcutaneous injection with OVCAR8 cells. Every 3 days, tumor volumes were monitored and calculated by the formula: *V* = (*L* × *W*2)/2 (*V*, volume; and *W*, width; *L*, length). Mice were randomly assigned into four groups with seven mice per group, when mice bearing tumors with mean volumes of 150 to 200. Group 1 received 0.2 mL DMSO as control; Group 2 was treated with 5 mg/kg of GSK2578215A, Group 3 was given 50 mg/kg of Olaparib, and Group 4 was treated with GSK2578215A and Olaparib. All treatments were performed by intraperitoneal injection. At day 28, the mice were euthanized and the major organs and tumor were excised, fixed, and sliced for H&E staining.

### 2.10 Immunohistochemistry analysis

Immunohistochemical staining was carried out following the manufacturer's instructions. Aperio software and Image J were used for imaging analysis. Three independent pathologists blinded to score all of the samples. The final intensity score was determined by majority vote. Each specimen was scored according to staining intensity (no staining: 0; slight staining: 1; moderate staining: 2; strong staining: 3) and staining area percentage (negative:0; <10% positive cells:1, 11‐50% positive cells:2, 51‐80% positive cells:3, >80% positive cells:4).

### 2.11 Statistics

All the data are represented as mean ± SEM. To compare differences between the experimental groups, a Student's bilateral *t*‐test was adopted. Statistical significance is represented in all indicators as follows *P < .05; **P < .01, ***P < .001, and n.s (not significance). *P* < .05 was supposed to be statistically significant.

## 3 RESULTS

### 3.1 A LRRK2 inhibitor GSK2578215A suppresses homologous recombination

**FIGURE 1:**
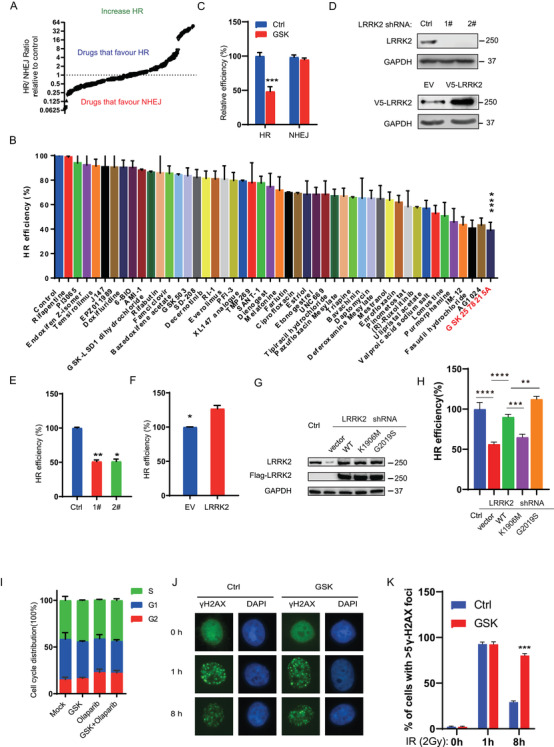
The LRRK2 inhibitor, GSK2578215A suppresses homologous recombination. (A) OVCAR8 cells harboring the SeeSaw 2.0 reporter system were treated with small molecule drugs as indicated. Drugs with an average normalized RFP:GFP ratio below 1were included in the category of drugs that decrease HR. In contrast, RFP:GFP ratio above 1 means that drugs favor HR. (B) HR efficiency was detected in drugs with RFP:GFP ratio below 1. (C) The HR/NHEJ efficiency of cells treated with GSK2578215A (1 μM for 24 h) was analyzed using HR/NHEJ reporter, respectively. (D‐F) The HR efficiency of OVCAR8 cells with LRRK2 knockdown (D, E) or OVCAR10 cells with LRRK2 overexpressing (D, F) was analyzed using the HR reporter assay. (G) Western blot analysis of LRRK2 or FLAG‐tag after transduced LRRK2 variants (including FLAG‐tagged WT, K1906M, G2019S) into LRRK2 knockdown OVCAR8 cells. (H) HR efficiency of OVCAR8 cells with LRRK2 knockdown and LRRK2 variants rescue. (I) Cell cycle analysis of OVCAR8 cells with or without GSK2578215A treatment (1 μM for 24 h) showed that GSK2578215A did not change cell cycle distribution of OVCAR8 cells. (J‐K) Representative images (J) and quantification (K) of γH2AX foci in control and GSK2578215A (1 μM)‐treated OVCAR8 cells with IR (2 Gy) treatment in different times. Data are shown as mean ± SEM from three independent experiments. *P*‐value was determined by two‐tailed unpaired *t*‐test (*, *P* < .05; **, *P* < .01; ***, *P* < .001; n.s., no significance)

HR plays an important part in error‐free DNA double‐strand break repair, thereby, maintaining genome stability. However, cancer cells have developed different kinds of mechanisms to upregulate HR and confer therapeutic resistance. Thus, inducing HRD is a viable strategy to sensitize cancer cells to DNA damaging therapies and overcome therapeutic resistance. To this end, we screened a library of small molecules to identify potential drugs that suppress HR using the SeeSaw 2.0 Reporter (SSR2.0) system. The SSR2.0 system is a novel tool to study the pathway of DNA double‐strand break repair, in which red fluorescent protein (RFP)‐positive and green fluorescent protein (GFP)‐positive cells represent the repair of I‐sce1‐induced DSBs by HR or NHEJ, respectively.^13^ OVCAR8 OC cells harboring the SSR2.0 system was treated with a library of small molecule drugs from TargetMOL (Catalog No. L8200), HR and NHEJ efficiency were then measured. Drugs were divided into two categories based on the average normalized RFP:GFP ratio. Drugs belong to the class that favor NHEJ with ratio below 1. On the contrary, drugs with an average RFP:GFP ratio above 1 were classified as sort of drugs that favor HR (Figure 1A). Detail of drugs is shown in Supporting information Table S1. The HR efficiency of drugs with an RFP:GFP ratio below 1 is presented in Figure 1B. Treatment with GSK2578215A (GSK)‐a, the LRRK2 inhibitor, resulting in the most significant decrease in HR efficiency, prompting us to focus on this target for our study. To further confirm the effect of GSK2578215A on HR, we employed the well‐established DR‐GFP reporter assay in the OVCAR8 OC cell line. As shown in Figure 1C, GSK2578215A treatment significantly inhibited HR activity but not NHEJ. To check whether GSK2578215A inhibits HR activity through LRRK2, we evaluated the protein expression of LRRK2 and phosphorylation of LRRK2 in several OC cell lines and found that OVCAR8 and OV56 cells have relatively high expression of LRRK2. OVKATE cells have moderate expression, and other cell lines, including A2780, PEO1, OV90, SKOV3, and OVCAR10, have low or undetectable expression of LRRK2. LRRK2 phosphorylation is correlated with expression levels (Supporting information Fig. S1). Next, we generated LRRK2 knockdown and overexpressing cells in OVCAR8 and OVCAR10 cells, respectively. The establishment of the cell lines was confirmed by Western blot (Figure 1D), and the effect of LRRK2 on HR was evaluated. As shown in Figure 1E and F, depletion of LRRK2 led to a dramatic decrease of HR efficiency in OVCAR8 cells, while LRRK2 overexpression promoted HR in OVCAR10 cells. To further establish the role of LRRK2 in HR, we transduced FLAG‐tagged and shRNA‐resistant LRRK2 variants (including WT, K1906M, G2019S) into LRRK2 knockdown OVCAR8 cells and checked the HR efficiency. We found that expression of WT and G2019S kinase active mutant can rescue the HR efficiency but a K1906M kinase dead mutant failed to do so (Figure 1G and H). Importantly, GSK treatment did not show an effect on cell cycle progression (Figure 1I). These results suggest that LRRK2 plays a great role in HR in an LRRK2‐kinase dependent manner. To further investigate the effect of GSK2578215A on DSB repair, cells were treated with GSK2578215A followed by IR treatment, and γH2AX foci, markers of DSB, were detected by immunofluorescence to determine whether GSK2578215A affects DDR and repair. As shown in Figure 1J and K, cells treated with GSK2578215A showed increased γH2AX foci at a later time point (8 h), suggesting that GSK2578215A impairs DNA repair. Together, these results demonstrate that LRRK2 is important for regulating HR, and LRRK2 inhibition suppresses HR, leading to accumulated DNA damage in cells.

### 3.2 LRRK2 inhibition enhances PARP inhibitor cytotoxicity in OC cells

**FIGURE 2:**
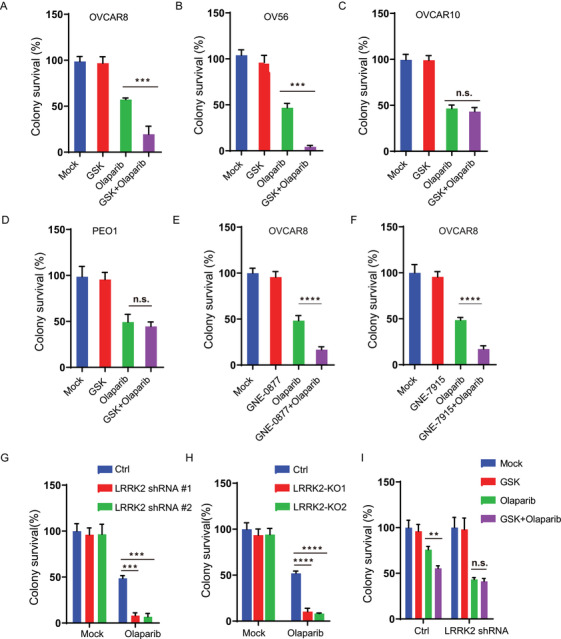
LRRK2 inhibition enhances PARP inhibitor cytotoxicity in ovarian cancer cells. (A‐C) Colony formation analysis of OVCAR8 (A), OV56 (B), and OVCAR10 (C) cells treated with control (Mock), GSK2578215A (GSK, 1 μM), Olaparib (0.8 μM), or their combination (GSK + Olaparib). (D) Colony formation analysis of PEO1 cells treated with control (Mock), GSK2578215A (GSK, 1 μM), Olaparib (0.3 μM), or their combination (GSK + Olaparib). (E‐F) Colony formation analysis of OVCAR8 cells treated with control (Mock), GNE‐0877 (1 μM), GNE‐7915 (1 μM), Olaparib (0.8 μM), or their combination (GNE‐0877 + Olaparib or GNE‐7915 + Olaparib). (G) Colony formation analysis of control and LRRK2 knockdown OVCAR8 cells treated with Olaparib (0.8 μM). (H) Colony formation analysis of control and LRRK2 knockout OVCAR8 cells treated with control (Mock) or Olaparib (0.8 μM). (I) Colony formation analysis of control and LRRK2 knockdown OVCAR8 cells treated with control (Mock), GSK2578215A (GSK, 1 μM), Olaparib (0.2 μM), or their combination (GSK + Olaparib). Data are shown as mean ± SEM from three independent experiments. *P*‐value was determined by two‐tailed unpaired *t*‐test (*, *P* < .05; **, *P* < .01; ***, *P* < .001; n.s., no significance)

Since GSK2578215A suppresses HR (Figure 1), we hypothesized that GSK2578215A could sensitize OC cells to PARP inhibitors. To test this, we analyze the effect of GSK2578215A on PARP inhibitor cytotoxicity in cells with high or low expression of LRRK2. As shown in Figure 2A–D, GSK2578215A treatment sensitized OVCAR8 and OV56 cells with high levels of LRRK2 to PARP, while single‐agent treatment with GSK2578215A had no obvious cytotoxic effect (Figure 2A and B). In contrast, GSK2578215A cannot potentiate PARP inhibitor cytotoxicity in OVCAR10 and PEO1 cells with low levels of LRRK2 (Figure 2C and D), suggesting that high expression of LRRK2 is required for the enhancement effect of GSK2578215A on PARP inhibitor cytotoxicity in OC cells. To further confirm that LRRK2 is required for the synergistic effect on PARP, we treated OVCAR8 cells with two other LRRK2‐specific inhibitors (GNE‐0877 and GNE‐7915) with or without PARP inhibitor. As shown in Figure 2E and F, we observed similar results. In line with these results, we found that compared to control group, knockdown or knockout of LRRK2 did not change cell viability but increased the sensitivity to PARP in OVCAR8 cells (Figure 2G and H). Moreover, GSK2578215A cannot sensitize LRRK2 knockdown cells to PARP inhibitor (Figure 2I), demonstrating that GSK2578215A regulates cellular sensitivity to PARP inhibitor in an LRRK2‐dependent manner. Collectively, our findings suggest that GSK2578215A may be a promising PARP inhibitor‐sensitizing agent in OC cells with high expression of LRRK2.

### 3.3 LRRK2 inhibition impedes the recruitment of RAD51 to DNA damage sites by disrupting the interaction of RAD51 and BRCA2

**FIGURE 3:**
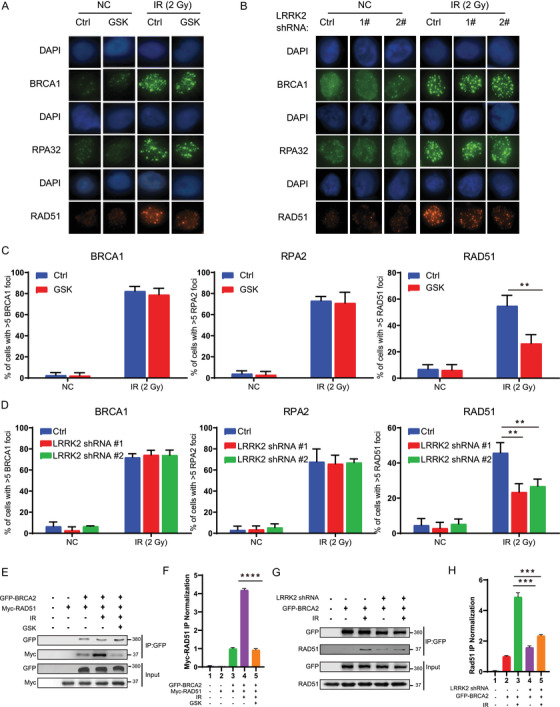
LRRK2 inhibition impedes the recruitment of RAD51 to DNA damage sites by disrupting the interaction of RAD51 and BRCA2. (A‐D) Representative images (A, B) and quantification (C, D) of BRCA1, RPA2, and RAD51 foci in OVCAR8 cells treated with GSK2578215A (A, C), or stably expressing LRRK2 shRNA (B, D) with or without IR treatment. (E) HEK293T cells were transfected with GFP‐BRCA2 and Myc‐RAD51, and then treated with GSK2578215A followed by IR treatment. The cells were lysed, and immunoprecipitated with anti‐GFP agarose beads. The beads were boiled and blotted with indicated antibodies. (G) HEK293T cells stably expressing control or LRRK2 shRNA were transfected with GFP‐BRCA2 followed by IR treatment. The cells were lysed, and immunoprecipitated with anti‐GFP agarose beads. The beads were blotted with indicated antibodies. (F, H) Quantification of relative expression of RAD51 according to E and G, respectively. Data are shown as mean ± SEM from five independent experiments. *P*‐value was determined by two‐tailed unpaired *t*‐test.*, *P* < .05; **, *P* < .01; ***, *P* < .001; n.s., no significance

Next, we wondered how LRRK2 inhibition suppresses HR. We examined the effect of GSK2578215A on the recruitment of core HR components (BRCA1, RPA2, and RAD51) to DNA damage sites by monitoring their foci formation using immunofluorescence analysis. As shown in Figure 3A, GSK2578215A treatment led to a significant decrease of RAD51 foci but not BRCA1 and RPA2 foci upon IR treatment in OVCAR8 cells. Similarly, decreased RAD51 foci induced by LRRK2 inhibition was also found following hydroxyurea and camptothecin treatment (Supporting information Fig. S2A–D). Additionally, we checked RAD51 foci in BRCA2 knockdown OVCAR8 cells to evaluate the relative impact of LRRK2 inhibition for disrupting of RAD51 activity following DNA damage. GSK2578215A treatment decreased RAD51 foci significantly, though not as dramatically as BRCA2 knockdown (Supporting information Fig. S2E–G) in response to DNA damage, suggesting that GSK2578215A impedes RAD51 recruited to DNA damage sites. Consistent with these results, a decrease of RAD51 but not BRCA1 and RPA2 foci was observed in LRRK2 knockdown OVCAR8 cells upon IR treatment (Figure 3B–D). This demonstrates that LRRK2 is required for recruiting RAD51 to the sites of DNA damage, and LRRK2 inhibition hinders this process, thereby suppressing HR. Next, we investigated how LRRK2 regulates the recruitment of RAD51 to the DNA damage sites. It is well known that the interaction between RAD51 and BRCA2 plays a great part in recruiting RAD51 to the sites of DNA damage and promoting DNA repair. We hypothesized that LRRK2 inhibition may impede the recruitment of RAD51 to the sites of DNA damage by disrupting the interaction of RAD51 and BRCA2. To test this, HEK293T cells were cotransfected with GFP‐BRCA2 and Myc‐RAD51, and then treated with GSK2578215A followed by IR treatment and immunoprecipitation assay. As shown in Figure 3E and F, the interaction of BRCA2 and RAD51 was induced by IR treatment in HEK293T cells untreated with GSK2578215A. In contrast, the IR‐induced interaction of RAD51 and BRCA2 was highly impaired in the GSK2578215A‐treated cells, and this interaction was also impaired upon hydroxyurea and camptothecin treatment (Supporting information Fig. S2H–K), suggesting that GSK2578215A hinders the association of RAD51 and BRCA2 following DNA damage. Besides, we also examined the interaction of RAD51 and BRCA2 in LRRK2 knockdown OVCAR8 cells, and we found that loss of LRRK2 highly reduced the interaction between RAD51 and BRCA2 induced after IR treatment (Figure 3G and H). These findings suggest that one mechanism for suppressing HR by the LRRK2 inhibitor GSK2578215A involves decreasing the interaction of RAD51 and BRCA2 which impedes the process of recruiting RAD51 to DNA damage sites.

### 3.4 Combination treatment with GSK2578215A and Olaparib synergistically suppresses OC cell viability in vivo

**FIGURE 4:**
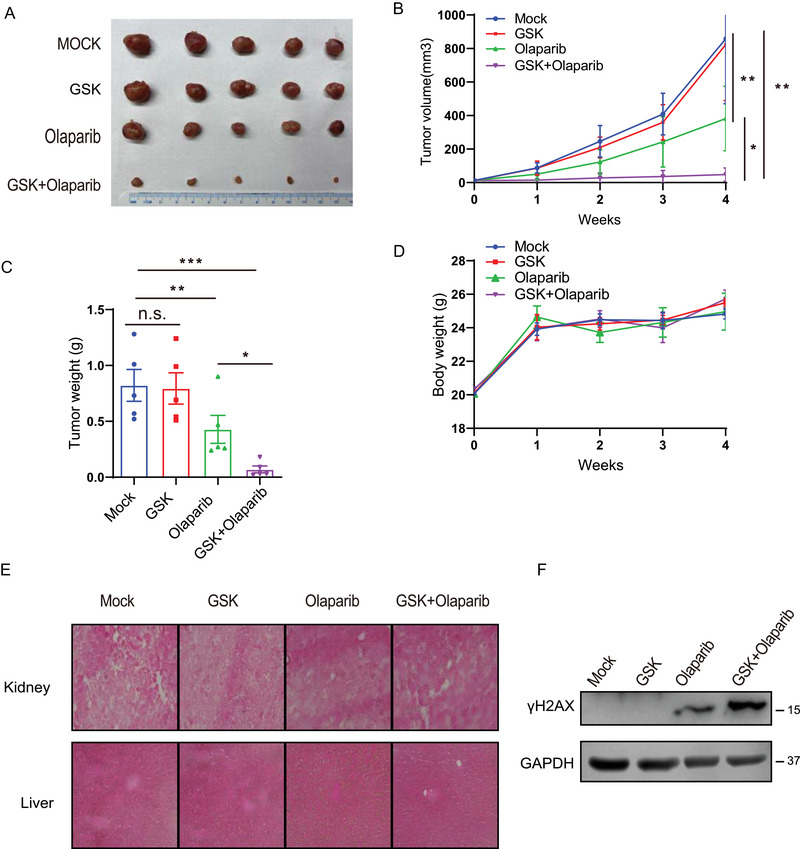
Combination therapy with GSK2578215A and Olaparib synergistically suppresses ovarian cancer growth in vivo. (A‐C) Mice bearing OVCAR8 xenografts were treated with vehicle control (MOCK), GSK2578215A (GSK, 5 mg/kg, T.I.W.), Olaparib (50 mg/kg/, T.I.W), or their combination (GSK + Olaparib). Representative tumor images (A), growth curve (B), and tumor weight (C) are shown. (D) Body weight of mice treated with control (Mock), 5 mg/kg GSK2578215A (GSK), 50 mg/kg Olaparib, or their combination. (E) Hematoxylin and eosin staining of liver and kidney organs after various treatments. (F) Western blot analysis of γH2AX levels in tumors of xenograft model. Data are shown as mean ± SEM from three independent experiments. *P*‐value was determined by two‐tailed unpaired *t*‐test. *, *P* < .05; **, *P* < .01; ***, *P* < .001; n.s., no significance

Above results demonstrate that GSK2578215A treatment suppresses HR and increases the susceptibility of OC cells to Olaparib, which prompted us to evaluate the effect of combination therapy with GSK2578215A and Olaparib in vivo. Nude mice bearing OVCAR8 cells xenografts were generated and treated with Olaparib (50 mg/kg, intraperitoneal injection, T.I.W for 3 weeks), GSK2578215A (5 mg/kg, intraperitoneal injection, T.I.W for 3 weeks), or Olaparib combined with GSK2578215A. As shown in Figure 4A–C, single GSK2578215A treatment did not show an obvious apparent effect on antitumor. Compared with Olaparib therapy alone, the combination therapy more effectively inhibited the growth of OC cells. Notably, the combination treatment showed no obvious effect on the weight of mice or toxicity in normal kidney and liver tissues (Figure 4D and E). Moreover, GSK2578215A treatment increased DNA damage induced by Olaparib in vivo (Figure 4F). Collectively, our data suggest that combination treatment of GSK2578215A and Olaparib may be a new treatment strategy for OCs with high expression of LRRK2.

### 3.5 High expression of LRRK2 is associated with advanced clinical factors and poor survival of OC patients

**FIGURE 5:**
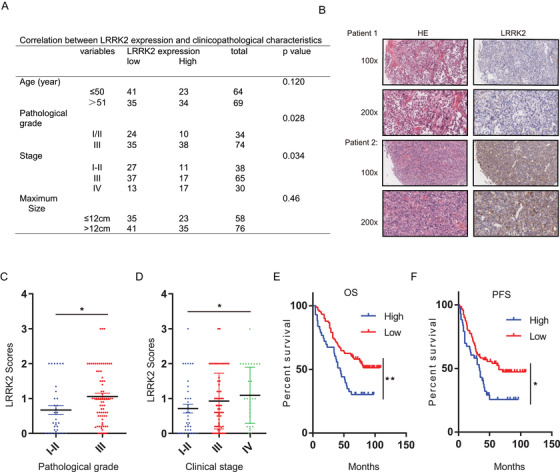
High expression of LRRK2 is associated with advanced clinical factors and poor survival of ovarian cancer patients. (A) The expression of LRRK2 in 133 ovarian cancer patients was examined by immunohistochemical (IHC) analysis. (B) Representative images of immunohistochemical staining of LRRK2 on tissue microarray of ovarian cancer specimens (n = 133). (C‐D) LRRK2 expression in different pathological grade (C) and clinical stage (D) of ovarian cancers was detected by IHC analysis. (E‐F) Overall survival (OS) and progression‐free survival (PFS) of ovarian cancer patients was analyzed by the Kaplan–Meier Plotter analysis. LRRK2 high: n = 57; LRRK2 low: n = 76. Data are shown as mean ± SEM from three independent experiments. *P*‐value was determined by two‐tailed unpaired *t*‐test (*, *P* < .05; **, *P* < .01; ***, *P* < .001; n.s., no significance)

Our data show that LRRK2 has an important role in promoting HR, and the LRRK2 inhibitor GSK2578215A sensitizes OC cells with high expression of LRRK2 to Olaparib in vitro and in vivo. Therefore, this is a great clinical value to evaluate the expression of LRRK2 in OCs. To this end, we collected 133 OC patient samples with detailed clinical information, including pathologic characteristic and survival prognosis (Figure 5A), and examined the expression of LRRK2 by IHC staining. The cut‐off value of the sum score of LRRK2 immunostaining was defined as 1.0. The immunostaining for LRRK2 was designated positive if the score was equal or greater than 1.0. As shown in Figure 5A and B, high expression of LRRK2 was detected in 57 cases, accounting for approximately 43% of OCs. Notably, high expression of LRRK2 is associated with worse pathological grade and a more advanced clinical stage (Figure 5C and D). In line with these results, compare to OC patients with low expression of LRRK2, those with high LRRK2 levels had poorer overall survival (OS) and progression‐free survival (PFS) (Figure 5E and F). Collectively, our results suggest that OCs expressing high levels of LRRK2 are more resistant to treatment potentially through promoting the HR pathway.

## 4 DISCUSSION

OC is one of the deadliest gynecologic malignancies in women. Therapeutic resistance and lack of prognostic and predictive molecular biomarkers have been persistent obstacles in the treatment of advanced OC. In recent years, PARP inhibitors have emerged as a new therapy for OCs for which there have been limited treatment options. However, cancer cells have developed multiple mechanisms to upregulate HR and confer therapeutic resistance to PARP inhibitor. Therefore, inducing HRD is a feasible way to sensitize cancer cells to DNA damaging therapies. Here, we screened a library of small molecules to identify potential drugs that suppresses HR and sensitizes cancer cells to DNA damaging therapy. We found that GSK2578215A, a LRRK2 inhibitor, suppresses HR and increases sensitivity of ovarian cancer cells to Olaparib in vitro and in vivo; suggesting that combination treatment of an LRRK2 inhibitor and a PARP inhibitor may be a novel strategy to improve the effectiveness of LRRK2‐ expression ovarian cancers.

**FIGURE 6:**
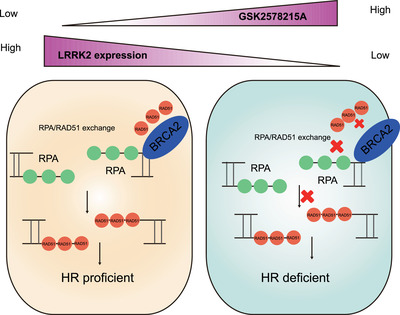
General model for LRRK2 protein function in HR. LRRK2 inhibition or depletion suppresses HR by impeding the recruitment of RAD51 to DNA damage sites via disrupting the interaction of RAD51 and BRCA2

LRRK2 is a multidomain protein containing GTP‐binding regulatory domain (ROC‐COR) and a kinase domain.^14^ Increased kinase activity of LRRK2 and hyperphosphorylation of LRRK2 kinase substrates are related to the pathological function of LRRK2 in disease.^15^ The kinase‐active G2019S mutation LRRK2 is a common cause responsible for familial PD.^10^, ^16^ It has been previously shown that the engineered K1906M mutation is effective in inhibiting LRRK2 kinase activity.^17^ Recently, accumulating evidences have proposed that LRRK2 mutations are highly associated with cancers. For example, PD patients bearing LRRK2 G2019S mutation have an increased risk of cancers, such as melanoma^18^ and hormone‐related cancer.^19^ In addition, comprehensive sequence‐based analysis suggests that LRRK2 may be mutated in OCs.^20^ To better understand the association between LRRK2 and cancers, Wu et al. knocked down LRRK2 and found that silencing LRRK2 suppresses thyroid cancer cell growth by facilitating apoptosis and cell‐cycle arrest.^21^ On the contrary, Liu et al^22^ showed that LRRK2 overexpression inhibits cell proliferation, migration, and invasion. However, our results showed that modulating expression of LRRK2 does not alter cell proliferation compared to control cells. It is unclear what causes these different results. One reason may be the different cell lines used. Notably, Chen tried to explore the primary physiological function of LRRK2 response to DNA damage and demonstrated that LRRK2 is a downstream substrate of Ataxia telangiectasia mutated (ATM) DNA damage, suggesting a potentially important role of LRRK2 in DDR and repair.^23^ But, to date, the molecular mechanism for LRRK2 response to DDR is still obscure. Here, we found that LRRK2 is required for the interaction of RAD51 and BRCA2, which is responsible for the recruitment of RAD51 to DNA damage sites in LRRK2–high‐expression cancer cells. Loss of LRRK2 resulted in decreased BRCA2‐RAD51 binding, thereby preventing RAD51 localization to DNA damage sites and suppressing HR. Moreover, we transduced LRRK2 variants such as a well‐known pathological dominant G2019S mutant that enhances kinase activity and the LRRK2 catalytic loss‐of‐function mutant K1906M, into LRRK2 knockdown OVCAR8 cells and checked the HR efficiency. We found that expression of WT and kinase active G2019S mutant could rescue the HR efficiency but the K1906M kinase dead mutant could not, indicating that the function of LRRK2 on HR is dependent on the kinase activity. Furthermore, our study shows that LRRK2 high expression is associated with poor survival, more advanced clinical stage, and high pathological grade of OC patients, highlighting that cancers expressing high levels of LRRK2 are more resistant to treatment potentially through promotion of the HR pathway. The loading of RAD51 to ssDNA is important for HR. Previous studies have revealed the mechanisms underlying the interaction of BRCA2 and RAD51. The BRC repeat domain of BRCA2 promotes RAD51 recruitment to ssDNA and accelerates RPA displacement of RPA by RAD51 in ssDNA.^24^ In addition, a study found that CDK activity may affect the interaction between C‐terminal domain of BRCA2 and RAD51 in cell cycle.^25^ A previous study also suggested that deubiquitination of RAD51 promotes the BRCA2‐RAD51 interaction and RAD51 recruitment.^26^ It is still unclear how LRRK2 promotes BRCA2‐RAD51 interaction. LRRK2 has preference for phosphorylating threonine and serine,^27^ and regulates protein‐protein interactions. Eguchi^28^ showed that Rab GTPases phosphorylated by LRRK2 repealed the interaction of Rab with GDI1/2, which is also reported by Steger.^29^ Therefore, we proposed that serine or threonine site of RAD51 or BRCA2 might be phosphorylated by LRRK2 and then affect their interaction (Figure 6). Different phosphorylation sites on serine, threonine, or tyrosine residues within RAD51 have been shown to be involved in DDR. RAD51 phosphorylation on S14 and then on T13 has been shown to be required for recruiting RAD51 to DNA damage sites.^30^ Similarly, phosphorylation of RAD51 on T309,^31^S192,^32^ Y315, and Y54 has been identified to be essential for RAD51 foci formation at DNA damage sites. Additionally, it is known that serine 3291 of BRCA2 (Ser3291) phosphorylation inhibits RAD51 binding to the C‐terminal domain of BRCA2.^25^ In our study, we treated OVCAR8 cells with IR or IR combined with GSK2578125A and found that GSK2578125A treatment had no additional effect on phosphorylation of BRCA2 at serine 3291 upon IR (data not shown), suggesting that the function of GSK2578125A is not dependent on serine 3291 phosphorylation. We also could not find RAD51 phosphorylation by LRRK2 (data not shown). Overall, further studies are needed to detail the underlying mechanisms by which LRRK2 is regulated following DNA damage and contributes to HR‐mediated DNA repair in cancers. Our study identifies LRRK2 as a new target that potentially regulates HR in ovarian cancer cells. This provides rationale for inhibiting LRRK2 to suppress HR in combination with PARP, thereby inducing synthetic lethality in cancer cells overexpressing LRRK2. However, two questions deserve further discussion. One concern is potential confounding roles of LRRK2 and PARP relative to mitochondrial DNA (mtDNA) damage. Previous reports showed that expression of LRRK2 G2019S mutation increases mtDNA damage. But D1994A mutant (kinase inactive) or wild type LRRK2 did not induce mtDNA damage in midbrain neuronal cultures.^33^ It may imply the function of LRRK2 in mtDNA damage is dependent on the G2019 mutation. In our study, to test the potential effect of GSK2578125A on mtDNA damage in ovarian cells, we screened ovarian cell lines and did not find G2019 mutation in OC cell lines (data not shown). Next, we quantified the mitochondrial membrane potential (ΔΨmito)—a indicator of mtDNA status using 3, 3′‐dihexyloxacarbocyanine iodide (DiOC6), the concentration of which in mitochondria is dependent on ΔΨmito.^34^, ^35^ Quantitative evaluation of cell fluorescence suggested that the mitochondrial membrane potential was not significantly changed after LRRK2 inhibition. These findings support the hypothesis that GSK2578125A has no effect on mtDNA damage. Another concern is DNA instability in the absence of LRRK2 activity. In our study, GSK2578215A sensitization to PARP is detectable only for ovarian cancer cell lines with LRRK2 overexpression. We did not see synergistic effect of GSK2578215A combined with Olaparib in LRRK2‐low cells. Furthermore, LRRK2 deficiency did not induce DNA instability and basal DNA damage. Therefore, we propose that LRRK2 is a regulator of HR, but is unlike essential factors, such as ATM or BRCA1, whose deficiency cause genomic instability even without external DNA damage. Taking the results together, we propose that the role of LRRK2 in HR is more evident in LRRK2‐high cells, and inhibition of LRRK2 in LRRK2‐high cells can increase susceptibility to DNA damage‐induced treatment. LRRK2 deficiency might not cause significant genomic instability in cells without external DNA damage, as the HR capability might be sufficient in these cells.

## CONFLICT OF INTEREST

The authors declare that they have no conflict of interest.

## ETHICS APPROVAL AND CONSENT TO PARTICIPATE

Animal experiments were completed respecting the approval of the Institutional Animal Care and Use Committee at Mayo Clinic (Rochester, MN).

## Supporting information

Supplementary Fig. 1: Western blot analysis of LRRK2 protein and phosphorylation status levels in ovarian cancer cell lines as indicated.

**Supplementary Fig. 2**: LRRK2 inhibition impedes the recruitment of RAD51 to DNA damage sites by disrupting the interaction of RAD51 and BRCA2

Supplementary Table 1. Original data obtained in the SSR screening. Drugs are ordered with HR/ENHJ ratio from high to low.

## Data Availability

The data generated during and/or analyzed during the current study are available from the corresponding author on reasonable request.
