# Supplementary figures and images for: LRRK2 inhibition potentiates PARP inhibitor cytotoxicity through inhibiting homologous recombination‐mediated DNA double strand break repair
Source: Clin Transl Med. 2021 Feb 26;11(3):e341. doi: 10.1002/ctm2.341 (PMC7908045; doi:10.1002/ctm2.341)

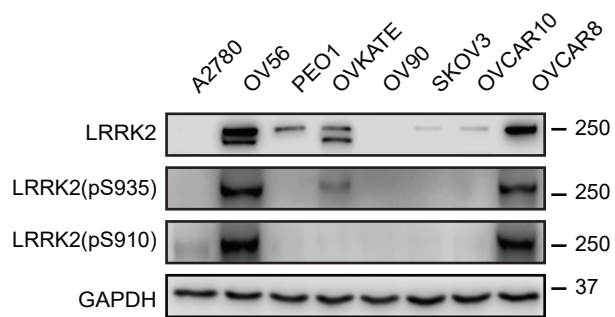

Supplement: Supplementary file 1 — Supplementary Fig. 1: Western blot analysis of LRRK2 protein and phosphorylation status levels in ovarian cancer cell lines as indicated. [file CTM2-11-e341-s001.pdf]

a

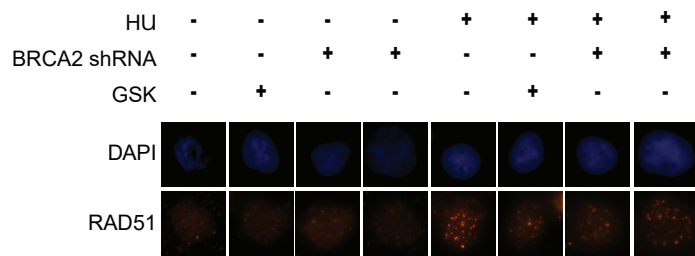

b

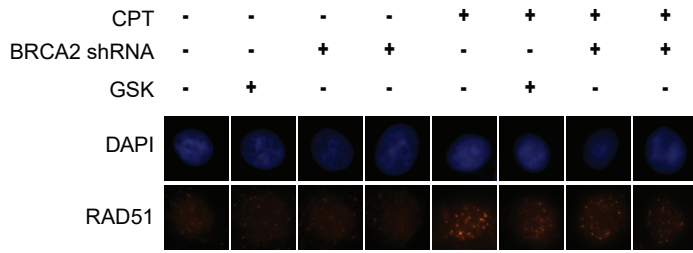

c

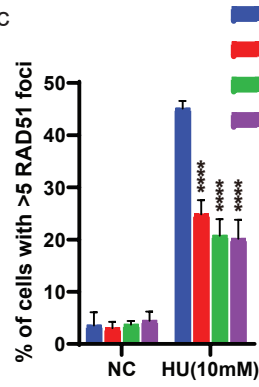

d

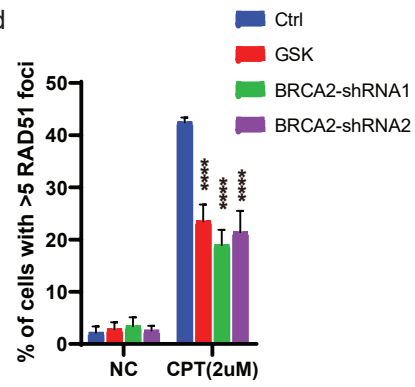

e

f

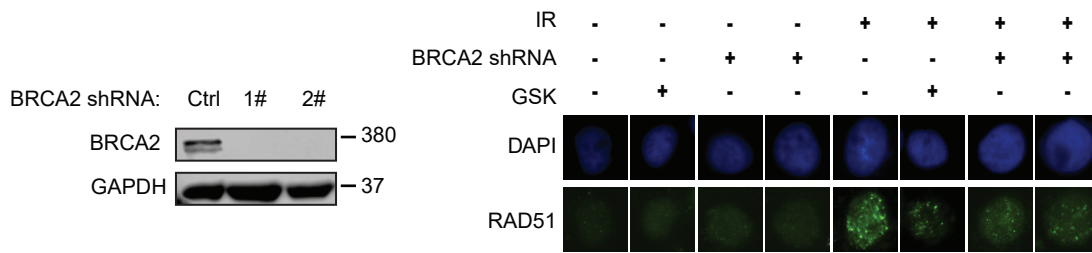

g

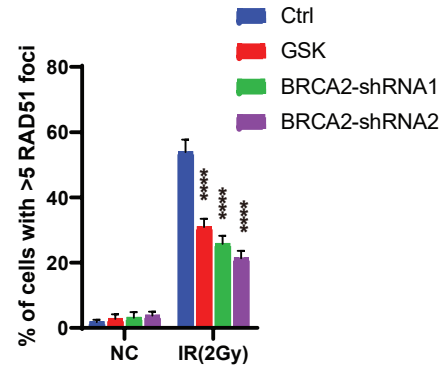

h

i

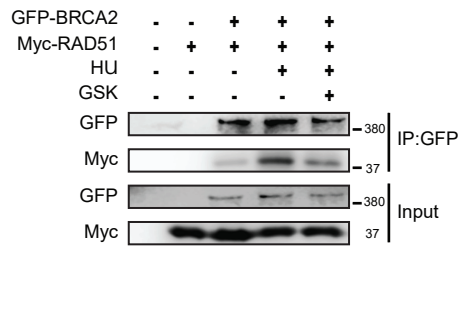

j

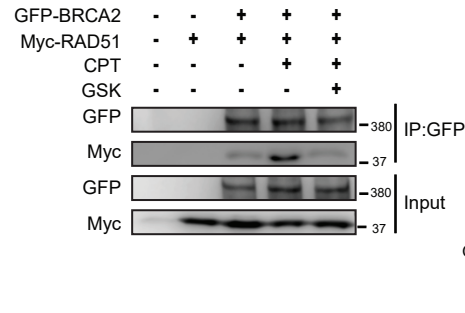

k

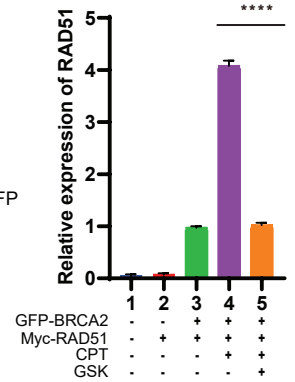

Supplement: Supplementary file 2 — Supplementary Fig. 2: LRRK2 inhibition impedes the recruitment of RAD51 to DNA damage sites by disrupting the interaction of RAD51 and BRCA2 [file CTM2-11-e341-s002.pdf]
